# Adipose-derived mesenchymal stem cells promote osteosarcoma proliferation and metastasis by activating the STAT3 pathway

**DOI:** 10.18632/oncotarget.15866

**Published:** 2017-03-03

**Authors:** Yan Wang, Yijing Chu, Bin Yue, Xuexiao Ma, Guoqing Zhang, Hongfei Xiang, Yong Liu, Tianrui Wang, Xiaolin Wu, Bohua Chen

**Affiliations:** ^1^ Department of Orthopaedic Surgery, the Affiliated Hospital of Qingdao University, Qingdao, China; ^2^ Department of Obstetrics and Gynecology, the Affiliated Hospital of Qingdao University, Qingdao, China

**Keywords:** osteosarcoma, adipose-derived mesenchymal stem cells, tumour microenvironment, signal transducer and activator of transcription 3, matrix metalloproteinase

## Abstract

Osteosarcoma is the most common primary bone malignancy in children and young adults, but the role of adipose-derived mesenchymal stem cells (ADSCs) in the rapid progression of osteosarcoma is still unclear. Here, we found that ADSCs promoted tumour growth and invasion by increasing matrix metalloproteinase 2/9 (MMP2/9) expression in tumour cells. The persistent activation of signal transducer and activator of transcription 3 (STAT3) has been shown to directly promote tumour growth by mediating a wide spectrum of cellular responses, and STAT3 activation was detected in osteosarcoma cells co-cultured with ADSCs or treated with ADSC-conditioned medium. Furthermore, siRNA-mediated STAT3 inhibition in osteosarcoma cells decreased cell proliferation and invasion and down-regulated MMP2/9 expression. In addition, a nude mouse model of osteosarcoma was established by injecting luciferase-labelled MG63 cells into the tibia. As shown in *in vivo* bioluminescence images, ADSCs promoted tumour cell proliferation, invasion progression and metastasis. STAT3 inhibition attenuated tumour growth and metastasis and prolonged the survival of these mice. After the siRNA treatment, the MMP2, MMP9 and Ki67 levels decreased. Based on these data, stromal ADSCs promote osteosarcoma progression by increasing STAT3 signalling-mediated MMP2/9 expression.

## INTRODUCTION

Osteosarcoma, the most common malignant bone tumour in children and adolescents, invades and destroys bone and adjacent soft tissues, and approximately 15% to 20% of patients will have clinically detectable metastases at presentation [1, 2]. Patients with metastatic disease at presentation have a worse prognosis. Currently, little is known about the role of adipose-derived stem cells (ADSCs) in the local aggression and distant metastasis of osteosarcoma; thus, further research regarding this phenomenon is required.

In addition to oncogenes and tumour suppressor genes, cross talk between tumour cells and their microenvironment also facilitates tumour growth and metastasis [3]. Stromal cells of haematopoietic or mesenchymal origin, together with the extracellular matrix and soluble factors, constitute a tumour microenvironment (TME) capable of facilitating tumour growth, angiogenesis, inflammation, and metastasis [4]. ADSCs isolated from the stromal vascular fraction of adipose tissue are able to differentiate into numerous cell lineages, including adipogenic, osteogenic, chondrogenic, myogenic and neurogenic cells [5]. Many important growth factors, cytokines, and chemokines secreted by ADSCs have been linked to cancer development and progression and result in more aggressive tumour behaviours [6, 7]. ADSCs make important contributions to the TME through a variety of mechanisms, but few studies have examined sarcomas originating from bone, cartilage, and muscle [8].

Signal transducer and activator of transcription 3 (STAT3), which mediates cellular responses to interleukins and many other growth factors, is aberrantly activated in the majority of cancers [9, 10]. The translocation of phosphorylated STAT3 mediates the up-regulation of key genes associated with tumourigenesis and tumour immune evasion, and genetic or epigenetic abnormalities resulting in elevated STAT3 expression/activation have been identified in a number of malignancies [11–13]. Additionally, mesenchymal stem cells (MSCs) in bone marrow promote osteosarcoma progression and protect tumour cells from drug-induced apoptosis through STAT3 signalling [14, 15].

Thus, we first examined ADSCs in the osteosarcoma microenvironment and determined how the properties of this environment affect tumour growth, migration, and metastasis. We used *in vitro* culture techniques to mimic this complex environment, and the activity of the STAT3 signalling pathway and matrix metalloproteinase (MMP) expression were examined to elucidate the mechanisms underlying these phenomena. Moreover, nude mice xenograft models were established, and tumour growth and metastasis were monitored using an *in vivo* imaging system. Taken together, our data indicate a relationship between ADSCs and osteosarcoma progression mediated by STAT3 signalling pathway activation, accompanied by increased MMP expression and decreased E-cadherin expression.

## RESULTS

### ADSC identification

**Figure 1:**
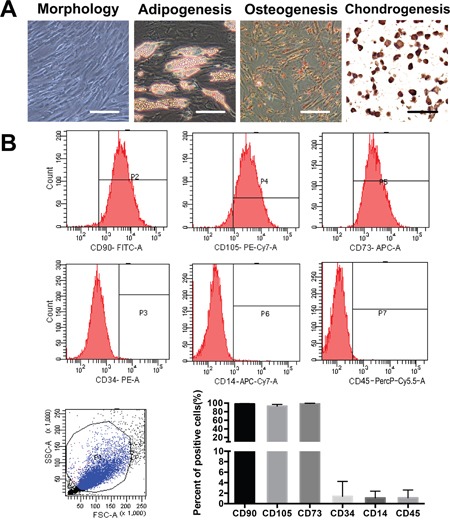
Isolation and characterization of ADSCs from adipose tissue surrounding the knee joint **(A)** Photomicrographs of primary ADSCs at passage 4 before and after achieving confluence are shown. The cells were examined for osteogenic, adipogenic, and chondrogenic differentiation. Scale bar=50 μm. **(B)** The purity of the isolated ADSCs was examined by flow cytometry; ADSCs express CD73, CD90 and CD105 and lack CD34, CD14 and CD45 expression. The quantification of three independent flow cytometry assays is shown.

Primary ADSCs acquired a fibroblastoid morphotype and successfully differentiated into osteoblasts, adipocytes and chondrocytes *in vitro* after several weeks of induction (Figure 1A). We examined the surface markers of three different ADSC cultures by flow cytometry and confirmed that the ADSCs expressed the appropriate positive markers (CD90, CD73 and CD105) and exhibited lower expression levels of the negative markers (CD34, CD45 and CD14) (Figure 1B).

### ADSCs trigger OS cell proliferation *in vitro*

**Figure 2:**
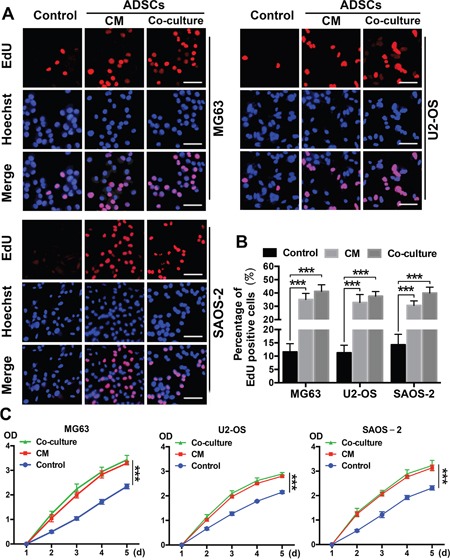
ADSCs trigger OS cell proliferation *in vitro* **(A)** Representative results of the EdU assay are shown. Dividing OS cells were labelled with EdU (red). All cells were counterstained with Hoechst 33342 (blue). **(B)** Quantitative analyses of the percentages of EdU-positive cells are shown below the images. Scale bar=25 μm. Error bars represent standard deviations. **(C)** The effects of ADSCs on OS cell proliferation were evaluated using the CCK8 assay. OS cells were treated with ADSC-conditioned medium, and the densities of both groups at 450 nm were analysed. Data from three separate experiments are shown. ***P<0.001.

We performed EdU and CCK-8 proliferation assays using OS cells that had been treated with ADSCs or ADSC-conditioned medium for 4 days to evaluate the ability of ADSCs to induce OS cell proliferation. OS cells cultured alone were used as controls. The ratio of EdU-labelled, actively proliferating OS cells (red) to total OS cells (blue) was calculated, and 3-5-fold increases in proliferation were observed in the ADSC and ADSC-conditioned medium groups (all P<0.001) (Figure 2A-2B) compared with the control group. Furthermore, no significant difference in the ratio of EdU-labelled cells to total cells was observed between the co-culture group and conditioned medium group. Similar results were observed in the CCK-8 experiments (Figure 2C).

### ADSCs trigger OS cell invasion by regulating MMP and E-cadherin expression

**Figure 3:**
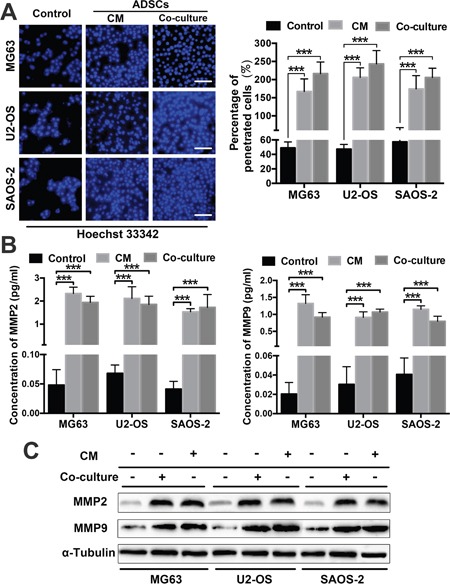
ADSCs trigger OS cell invasion by regulating MMP2/9 and E-cadherin expression **(A)** The number of OS cells that migrated through the 8-μm-pore transwell membrane were counted to determine the changes in OS cell invasion after treatment with ADSCs. **(B)** ELISAs were used to examine MMP2 and MMP9 expression in OS cell supernatants after co-culture or treatment with the conditioned medium. The supernatants of three OS cell lines cultured alone served as controls. **(C)** MMP2/9 and E-cadherin expression in ADSC-treated OS cells was examined by western blotting. * P<0.05, ** P<0.01, *** P<0.001.

We performed transwell invasion assays involving the three OS cell lines that were co-cultured with ADSCs or treated with ADSC-conditioned medium to further assess whether ADSCs increase OS cell aggression. OS cells cultured alone served as a control. The numbers of invading OS cells were increased in the group co-cultured with ADSCs and the group treated with ADSC-conditioned medium compared with the control group; 5-8-fold increases were observed (all P<0.001) (Figure 3A).

By degrading the extracellular matrix, MMPs pave the way for tumour cell invasion and metastasis [16, 17]. E-cadherin is involved in cell adhesion/polarity and tissue morphogenesis, and normal epithelial cells acquire invasive and migratory properties when they express lower levels of E-cadherin [18]. We sought to determine whether MMPs and E-cadherin play a role in the tumour-promoting effects of ADSCs. We detected the concentrations of secreted MMP2 and MMP9 in OS cell supernatants using ELISAs. The MMP2 and MMP9 levels were both significantly increased in the co-culture and conditioned medium groups (Figure 3B). We detected MMP2/9 and E-cadherin expression in OS cells treated with ADSCs or ADSC-conditioned medium by western blotting and found that the OS cells co-cultured with ADSCs and the cells treated with ADSC-conditioned medium exhibited a significant increase in MMP2 and MMP9 expression and a decrease in E-cadherin expression (Figure 3C).

### ADSCs activate STAT3 in OS cells

**Figure 4:**
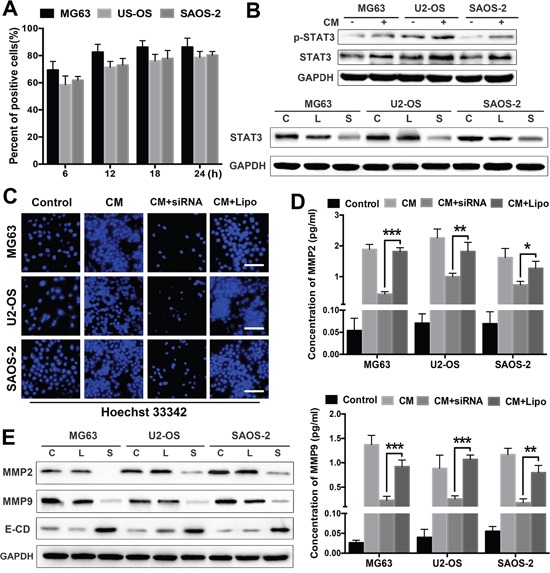
STAT3 signalling mediates the tumour invasion-promoting effects of ADSCs **(A)** The transfection efficiency of the siRNA was analysed in OS cells at different time points after transfection using flow cytometry. **(B)** Western blot analysis of STAT3 and pSTAT3 expression in OS cells treated with ADSC-conditioned medium. OS cells were pretreated with the STAT3 siRNA (50 nM) for 48 h before treatment with ADSC-conditioned medium for an additional 12 h. STAT3 expression levels were detected by western blotting. OS cells treated with Lipofectamine 2000 alone served as negative controls. **(C)** OS cell invasion was analysed using transwell assay. OS cells were transfected with the siRNA and treated with ADSC-conditioned medium. OS cells treated with ADSC-conditioned medium alone served as positive controls, and OS cells treated with Lipofectamine 2000 alone served as negative controls. **(D)** The MMP2 and MMP9 levels in the supernatants were detected using ELISAs. **(E)** OS cells were treated with the STAT3 siRNA or ADSC-conditioned medium, and MMP2/9 and E-cadherin expression in OS cells was examined by western blotting. OS cells treated with Lipofectamine 2000 alone served as negative controls. The results are expressed as mean±SD. Abbreviation: C Control; L Lipofectamine 2000; S siRNA. * P<0.05, ** P<0.01, *** P<0.001.

We examined the expression of the well-studied oncogenic protein STAT3 in OS cells treated with ADSCs to further elucidate the mechanisms underlying the OS-promoting effects of ADSCs. First, STAT3 activation in OS cells was inhibited by a specific siRNA targeting STAT3. The transfection efficiency of the siRNA was measured in OS cells at different time points after transfection using flow cytometry (Figure 4A). The transfection efficiencies of three lines of OS cells were greater than 70% at 18 h after transfection. We then examined the responses of OS cells to ADSCs by observing the effects of ADSC-conditioned medium on STAT3 expression. STAT3/pSTAT3 expression was detected in OS cells treated with ADSC-conditioned medium using western blotting. We observed distinct increases in STAT3/pSTAT3 expression in all three OS cell lines after 2 days (Figure 4B). Our western blotting results confirmed that 50 nM siRNA inhibited STAT3 activation in OS cells (Figure 4B).

### ADSCs promote OS cell invasion and proliferation by activating STAT3

Next, we examined the effects of STAT3 on the tumour invasion-promoting effects of ADSCs. OS cell invasion was significantly increased after treatment with ADSC-conditioned medium and was reduced in the cells transfected with the siRNA (Figure 4C). Furthermore, STAT3 inhibition dramatically suppressed MMP2 and MMP9 expression in OS cell supernatants treated with ADSC-conditioned medium (Figure 4D). We also evaluated the expression levels of MMP2, MMP9 and E-cadherin in siRNA-treated OS cells by western blotting, and STAT3 inhibition significantly decreased MMP2 and MMP9 expression in OS cells and increased E-cadherin expression (Figure 4E).

**Figure 5:**
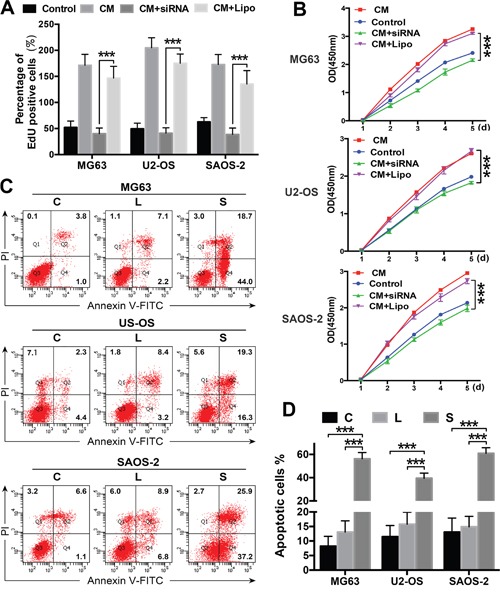
ADSCs promote cell proliferation and regulate apoptosis via STAT3 signalling **(A-B)** Cell proliferation was evaluated using the CCK8 assay and EdU analysis. OS cells were transfected with the siRNA and treated with ADSC-conditioned medium. OS cells treated with ADSC-conditioned medium alone served as positive controls, and OS cells treated with Lipofectamine 2000 alone served as negative controls. **(C-D)** OS cells were transfected with the STAT3 siRNA or treated with ADSC-conditioned medium, and apoptosis rates were determined using flow cytometry. OS cells treated with Lipofectamine 2000 alone served as negative controls. The percentages of Annexin V-positive cells are presented in bar charts. *** P<0.001.

OS cells were exposed to ADSC-conditioned medium, and their proliferation was assessed using CCK8 and EdU assays to determine whether ADSCs promote OS cell proliferation via STAT3 signalling. ADSC-conditioned medium increased OS cell proliferation, and STAT3 inhibition suppressed OS cell proliferation, even in the presence of the ADSC-conditioned medium (Figure 5A-5B).

STAT3 inhibition stunts tumour growth by inducing cell apoptosis in many types of malignant tumours [19, 20]. OS cell apoptosis was examined to investigate the mechanism underlying the effects of the STAT3 siRNA treatment. The number of apoptotic OS cells was significantly greater in the siRNA-transfected cells than in the control cells (Figure 5C-5D). Based on these data, ADSCs promote OS cell proliferation and invasion through the STAT3 signalling pathway by regulating apoptosis and MMP/E-cadherin expression in OS cells.

### ADSCs promote OS cell proliferation and metastasis in nude mouse models

**Figure 6:**
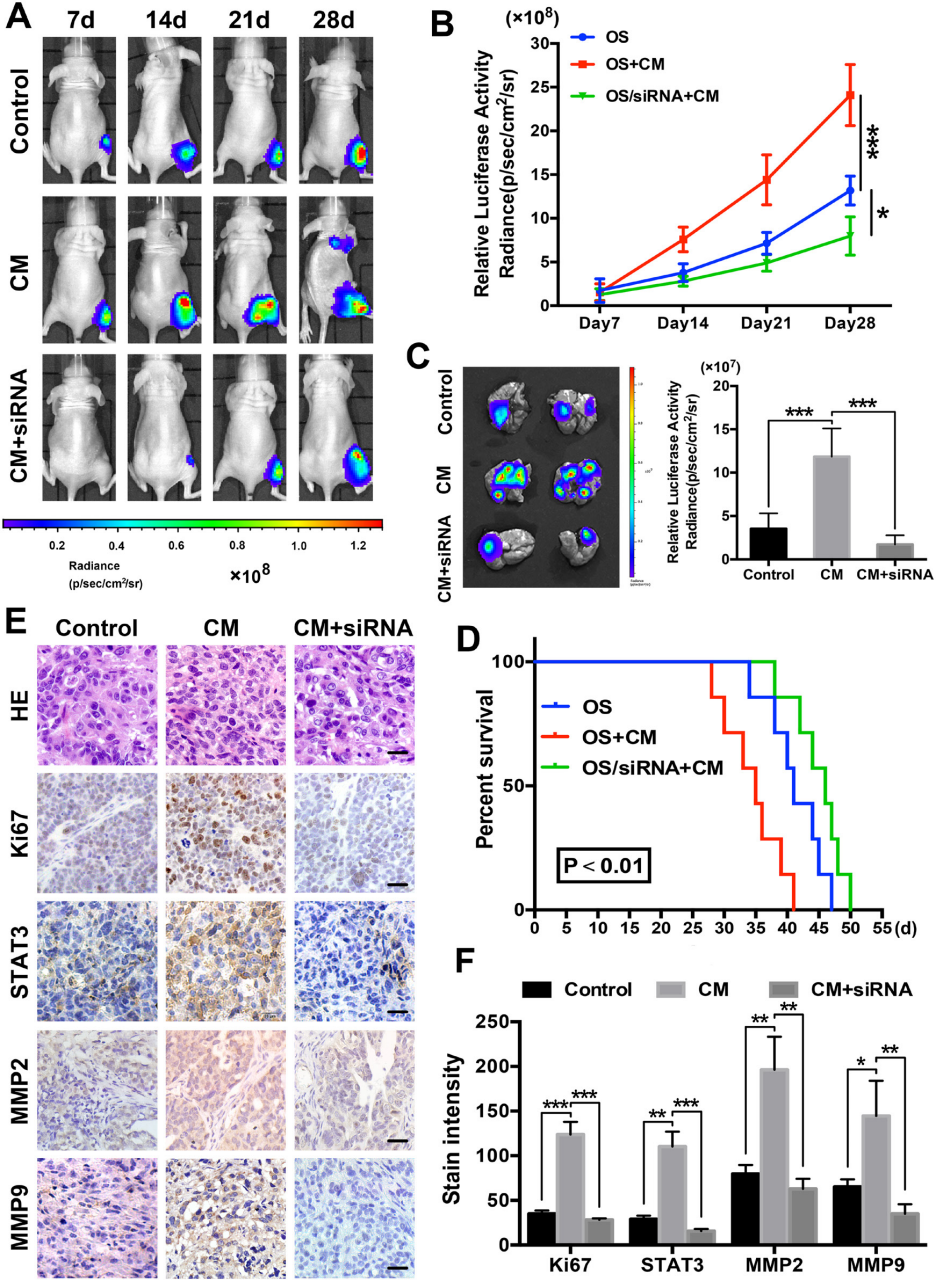
ADSCs promote osteosarcoma growth and metastasis in nude mouse models **(A)** An *in vivo* imaging system was used to monitor OS xenograft luminescence activity, which represented tumour growth and metastasis. **(B)** Living Image Software was used to analyse tumour bioluminescence intensity weekly. The quantitation of the normalized image counts is shown. **(C)** Lungs of the tumour-bearing mice were excised, and the bioluminescence intensity was analysed to determine the level of tumour metastasis in the lungs. **(D)** Survival curves of the three groups are shown, and the median survival of the OS group was 43 days, which was significantly longer than the survival of the OS + conditioned-medium group (25 days, P<0.01). **(E)** The immunohistochemical analysis of Ki67, STAT3, MMP2 and MMP9 expression in the orthotopic tumour xenografts is shown. **(F)** Quantitation of the intensity of Ki67, STAT3 and MMP2/9 staining in the xenografts. Scale bar: 25 μm. * P<0.05, ** P<0.01, *** P<0.001.

We established tibia osteosarcoma models in nude mice using human OS MG63 cells to determine the contributions of ADSCs and STAT3 to osteosarcoma growth and metastasis *in vivo*. STAT3 inhibition was facilitated by twice-weekly intra-tumour injections of the siRNA. Luciferase activity was measured using an IVIS imaging system to monitor the tumour cell volume. The signal increased more rapidly in the conditioned medium-treated group and more slowly in the siRNA-treated group than in the control group (Figure 6A-6B). Moreover, on day 28, we observed lung metastasis signals in the conditioned medium-treated group. We collected the animals’ lungs after sacrifice and immediately examined the bioluminescence to further evaluate the actual levels of metastasis. Lungs from the conditioned medium-treated group exhibited significantly higher luminescence signals, and the siRNA-treated group and control group showed similar signal intensities (Figure 6C). According to the survival curves, STAT3 inhibition prolonged the survival of mice with osteosarcoma; however, ADSC-conditioned medium decreased the survival of the animals (Figure 6D).

Finally, higher Ki67 levels were observed in the tumour tissues of the ADSC-conditioned medium group, suggesting that these tumours grew more rapidly. Higher STAT3, MMP2 and MMP9 levels were detected in the ADSC-conditioned medium group, and STAT3 pathway inhibition significantly reduced these levels (Figure 6E-6F). Thus, ADSCs promote osteosarcoma growth and metastasis and STAT3 pathway inhibition counteracts these effects.

## DISCUSSION

Accumulating evidence indicates that cancer cells themselves do not cause disease. Rather, cancer cells establish autocrine networks that produce a microenvironment that fuels disease progression [4, 21]. Thus, the TME plays a critical role in cancer initiation and progression. Adipose tissue has been increasingly recognized as the largest endocrine organ in the body, and adipose tissue-derived cells, such as cancer-related adipocytes and ADSCs, are recruited to cancer microenvironments to enhance protumour effects [22–24]. As shown in our previous study, omental ADSCs promote ovarian cancer cell proliferation and metastasis and are associated with the formation of pre-metastasis niches in the omentum [25]. As osteosarcoma is the most common primary malignant bone tumour in children and young adults, various tumour studies have focused on its biological characteristics, but its TME has rarely been studied. Late local osteosarcoma recurrence after autologous fat grafts has been observed, and local recurrence is most likely to occur in the soft tissues, indicating that fat tissues promote tumour growth [26, 27]. ADSCs are detected in perivascular adipose tissue [28], subcutaneous adipose tissue [29, 30], and intermuscular adipose tissue adjacent to osteosarcoma masses [31]; however, the relationship between ADSCs and osteosarcoma is still unclear.

In our study, we isolated ADSCs from adipose tissue adjacent to the knee joint to determine the role of ADSCs in multiple aspects of osteosarcoma development. ADSCs exerted paracrine-like effects on OS cells and activated the STAT3 signalling pathway in these cells. The STAT3 signalling pathway mediated OS cell proliferation and invasion and facilitated increases in MMP2/9 expression and decreases in E-cadherin expression. Furthermore, STAT3 activation was critical for osteosarcoma cell proliferation and survival in mouse xenograft osteosarcoma models. Therefore, ADSC-induced STAT3 activation in OS cells may play a central role in the penetration of osteosarcoma into neighbouring soft tissue.

Although cancer cell migration and metastasis clearly exert lethal effects, the exact mechanisms underlying these effects are still poorly understood. The TME plays an integral role in tumour anatomy and physiology [32]. Through interactions with the TME, ADSCs facilitate the progression of a variety of solid malignancies, including tumours of the breast, prostate, lung, ovary and pancreas [8, 33]. Several mechanisms by which ADSCs interact with cancer cells and influence their microenvironment have been proposed, including paracrine signalling and cell-to-cell signalling, as well as differentiation into cancer-associated myofibroblasts (CAFs) [34–37]. Thus, we hypothesize that ADSCs in adipose tissue interact with neighbouring osteosarcoma cells and stimulate disease progression. In this study, conditioned medium treatment and ADSC co-culture increased tumour growth, indicating that ADSCs may contribute to the formation of the TME within adipose tissue surrounding tumour masses.

STAT3 is a pro-oncogenic transcription factor and is normally strictly regulated by a tightly coordinated network of activators and deactivators. Based on emerging evidence, constitutive STAT3 activation in human malignancies is associated with tumour cell proliferation, survival and angiogenesis [38]. Constitutive STAT3 pathway activation was recently observed in osteosarcoma. Moreover, STAT3 inhibition plays a role in osteosarcoma cell proliferation, survival and migration [39]. Thus, we sought to identify the link between STAT3 activation by ADSCs in the TME and the osteosarcoma-promoting effects of ADSCs. The STAT3 signalling pathway was activated in OS cells exposed to the paracrine effects of ADSCs. The use of a specific siRNA to inhibit STAT3 expression in OS cells weakened the effects of ADSCs on osteosarcoma cell proliferation and invasion. Changes in apoptosis and the ratio of MMP/E-cadherin expression were observed in the siRNA-transfected cells. Thus, the interaction between ADSCs and osteosarcoma cells is mediated, at least in part, by the STAT3 pathway.

As important regulators of the tumour stroma, MMPs mediate crosstalk between the noncellular and cellular components of the TME to enhance tumour progression [40, 41]. In addition, the STAT3 signalling pathway regulates tumour invasion and metastasis by regulating MMP2 expression [42]. In our study, substantial increases in MMP2/9 expression were observed in supernatants from OS cells treated with ADSCs, and the expression of these proteins within OS cells was also elevated. Stable MMP2/9 up-regulation was observed in MG63 cells treated with ADSC-conditioned medium, indicating that MMP2/9 are associated with tumour promotion *in vitro* and *in vivo*.

Our study identified a signalling network involving ADSCs, adjacent joints and OS cells and determined that STAT3 is the primary mediator of this interaction. STAT3 activation is essential for the osteosarcoma-promoting effects of ADSCs. Consequently, ADSCs in adipose tissue may increase osteosarcoma cell proliferation and metastasis in conjunction with changes in MMP2/9 and E-cadherin expression, which are at least partially mediated by the activation of the STAT3 signalling pathway.

## MATERIALS AND METHODS

### ADSC isolation and culture

Adipose tissue adjacent to the knee joint was processed from two adult male donors (Age: 35y/40y; BMI: 26.21/28.05) who underwent open reduction and internal fixation for patellar fracture. All donors provided written informed consent, and this study was conducted according to institutional guidelines and an approved protocol. ADSCs were isolated as described [5]. Briefly, fresh adipose tissues were collected, washed with sterile phosphate-buffered saline (PBS) and minced into small pieces using scalpels. These pieces were then incubated with 0.1% collagenase (type I; Sigma-Aldrich, St. Louis, MO) in DMEM/F12 (Gibco, Grand Island, USA) for 1 h at 37°C. Then, the tissues were digested by collagenase and added to an equal volume of DMEM/F12 with 10% foetal bovine serum (FBS, Gibco, Australia) to inactivate the digestive fluid. This reaction mixture was then filtered through a 100-μm mesh filter, after which the filtrate was centrifuged, and the cells were plated onto culture plates and incubated in the indicated culture medium (DMEM/F12, 10% FBS) at 37°C in a CO_2_ incubator.

### ADSC characterization

ADSCs (passage 3-6) were analysed via flow cytometry with respect to cellular membrane marker expression using CD105-PECy7, CD73-APC, CD90-FITC, CD34-PE, CD14-APC-Cy7 and CD45-PerCP-Cy5 antibodies (all from eBioscience, San Diego, CA) [43].

The capacity of ADSCs to differentiate into osteoblasts, adipocytes and chondrocytes was assessed as described [44, 45]. ADSCs were treated with an Adipogenesis, Osteogenesis and Chondrogenesis Differentiation Kit (Gibco, Invitrogen Corporation, Carlsbad, CA). The medium was changed twice per week. After 3 weeks of differentiation, the ADSCs were stained with Oil Red O and Alizarin Red S. After 4 weeks of differentiation, immunohistochemical staining for type X collagen was performed. The results were recorded using an Olympus FV500 optical microscope (Olympus, Tokyo, Japan).

### Cell lines and culture conditions

Human OS SAOS-2 cells were purchased from the Chinese Academy of Sciences (Shanghai, China), and MG63 and U2-OS cells were obtained from the China Center for Type Culture Collection. MG63 and U2-OS cells were cultured in DMEM/F12 cultural medium with 10% FBS (Thermo Fisher, Waltham, MA). SAOS-2 cells were cultured in McCoy's 5A medium with 15% FBS (Thermo Fisher, Waltham, MA). All cells were grown in a humidified 5% CO_2_ incubator at 37°C.

### siRNA transfection

OS cells were seeded in 10-cm plates at a density of 5×10^5^ for 24 h to reach sub-confluence and then transfected with STAT3-specific siRNA or a scrambled siRNA control (Santa Cruz Biotechnology) using Lipofectamine 2000 (Invitrogen, Carlsbad, CA).

### Conditioned media

ADSCs were grown to near confluence in DMEM/F12 medium supplemented with 10% FBS, washed twice and cultured overnight in serum-free medium, which was then collected and frozen (−20°C). This medium was ADSC-conditioned medium (CM).

### Indirect co-culture

OS cells labelled with tracking dye (CFSE, V12883; Invitrogen, Carlsbad, CA) were mixed with ADSCs at a ratio of 1:4 (ADSCs 0.2×10^5^ cells, OS cells 0.8×10^5^ cells) and cultured in six-well plates for at least 3 days. Transwell plates (Corning, Life Sciences, Amsterdam, the Netherlands) with 0.4-μm-pore polycarbonate membranes were used for the indirect co-cultures, and the ratio of primary cells to OS cells was similar to that of the direct co-cultures. OS cells and ADSCs cultured alone served as negative controls.

### Invasion assay

Hoechst 33342 (Invitrogen, Carlsbad, CA)-labelled OS (5×10^5^) cells were plated on 24-well transwell plates (corning, NY, USA) with 8-μm-pore membranes coated with 50 μL of BD Matrigel™ Matrix (1:8 dilution). The lower chamber was filled with 600 μL of culture medium containing 10% FBS (control), 10% FBS and ADSCs (indirect co-culture) or ADSC-conditioned medium. Then, the transwell plates were incubated at 37°C for 6 h, and the cells on the lower surface of the membrane were fixed with 4% paraformaldehyde. All cells were cultured in medium without FBS for one day prior to the experiments to prevent differences in cell proliferation. The number of migrated cells per high-power field represented the invasive capability of the OS cells. All assays were performed in triplicate.

### Enzyme-linked immunosorbent assay

OS cell lines (0.2×10^5^ cells per well) were cultured in six-well plates overnight with culture medium containing 10% FBS. The cell supernatants were then placed in fresh culture medium without FBS and co-cultured indirectly with ADSCs in transwell plates with 0.4-μm pores for 2, 3 and 4 d. Then, MMP2 and MMP9 expression in the supernatant was measured via enzyme-linked immunosorbent assay (ELISA) (Raybiotech, GA, USA), according to the manufacturer's instructions.

### Western blotting

Cells were lysed in NP40 buffer (Beyotime, Shanghai, China) for 10 min on ice and then centrifuged at 10,000 g at 4°C to remove cell debris. Equal amounts (30 μg) of cell extract were resolved by SDS-PAGE and transferred to a PVDF membrane (Bio-Rad, Hercules, CA), followed by incubation with primary rabbit monoclonal antibodies against human STAT3 and pSTAT3 (1:1000 dilution; R&D Systems) and rabbit monoclonal antibodies against human MMP2/9, E-cadherin and α-tubulin (1:1000 dilution; ProteinTech, Chicago, IL). Then, the cells were incubated with peroxidase-conjugated affinipure secondary IgG antibodies (H+L) (1:2000; ProteinTech, Chicago, IL). Proteins were detected using a chemiluminescence detection system, and bands were quantitated by Image Lab™ Software, Version 5.1 (both from Bio-Rad, Hercules, CA).

### EdU proliferation analysis

We investigated the proliferation of OS cells and OS cells treated with conditioned medium or indirect co-culture for 4 days using a Cell-Light 5-Ethynyl-2′- deoxyuridine (EdU) [46] Cell Proliferation Kit (Guangzhou RiboBio, Guangzhou, China), according to the manufacturer's instructions. Briefly, OS cells were seeded in a 96-well plate, and the medium was replaced with 100 μL of 10 μM EdU medium in each well. The cells were then incubated for 2 h and fixed with 4% paraformaldehyde. The formaldehyde was neutralized with 2 mg/mL glycine solution, after which the cells were subjected to 0.5% Triton X-100 permeabilization. Then, the cells were stained with Apollo® 567 and incubated for 30 min before being permeabilized with 0.5% Triton X-100. The cells were subsequently counterstained with Hoechst 33342 and imaged via fluorescence microscopy. EdU-labelled cells in each well were counted manually in ten randomly selected fields of view, and the percentages of EdU-positive cells were used to determine cell proliferative activity. All assays were performed in triplicate.

### CCK8 cell proliferation assay

Cell proliferation was also measured using CCK-8 reagent (Dojindo, Japan). OS cells (5000 cells/well, 5 wells/group) were allowed to grow in 96-well plates. Cell proliferation was documented every day, in accordance with the manufacturer's protocol. CCK-8 reagent was added to each well 1.5 h before the end of incubation. The absorbance (OD value) was measured with a microplate reader at a wavelength of 450 nm. Colorimetric assay was performed, and growth curves were calculated using the mean results from three independent experiments.

### Apoptosis assay

OS cell apoptosis was quantified with an Annexin V-FITC apoptosis detection kit (BD Biosciences, San Diego, CA, USA) according to the manufacturer's instructions. Briefly, OS cells, Lipofectamine 2000- or siRNA-treated OS cells were washed with PBS and resuspended in binding buffer containing annexin V and propidium iodide (PI). Fluorescence intensity was measured using flow cytometry (BD Biosciences).

### Animals and xenograft model

Age-matched male BALB/c-nu nude mice between 3 and 4 weeks old (Beijing Vital River, China) were housed in a specific pathogen-free environment in the Experimental Animal Center of the Affiliated Hospital of Qingdao University, China. MG63 cells (8×10^6^ cells/mouse) were injected into the right proximal tibia to establish an OS xenograft model. ADSC-conditioned medium (twice per week) or the STAT3 siRNA (once per week) was injected into the tumour one week after the MG63 cell injection. Each of three groups included ten mice, which were injected with MG63 cells, MG63 cells + conditioned medium, or MG63 cells + siRNA + conditioned medium. The animals were sacrificed 50 days after cell injection, and the tumours were dissected, collected and weighed. The mice were handled and cared for according to the guidelines and protocols approved by the Animal Care and Use Committee of Qingdao Medical College.

The luminescence of the OS cells injected into the tibia marrow cavitywas monitored weekly using an *in vivo* imaging system (IVIS, Xenogen, Alameda, CA). All mice were imaged weekly after injection to monitor tumour growth. Animal weights were measured every 3 days until the end of the experiment. Next, for ex vivo bioluminescence imaging, the lungs were harvested and immediately subjected to imaging using the same conditions and system used for *in vivo* imaging. Living Image Software (Lumina II Living Image 4.3) was used to analyse the tumour bioluminescence intensity, and the total counts were normalized to the image acquisition time (photons/sec) and summed for the entire tumour area.

### Immunohistochemistry

Tissues from the xenograft tumours were fixed for 24 h and cut into 4-μm sections after being embedded in paraffin. After the tissues were processed with a graded series of alcohols, antigen retrieval was performed in 0.01 M citrate buffer (pH = 6.0) at 95°C for 20 min. Then, the cells were incubated in 3% H_2_O_2_ for 20 min to inactivate endogenous peroxidase and blocked in 5% BSA for 20 min at room temperature. Antibody staining with 100 μL of specific antibodies against human STAT3 (1:100 dilution; R&D Systems) and rabbit monoclonal antibodies against human Ki67, MMP2 and MMP9 (1:100 dilution; ProteinTech groups inc, Chicago, IL) was applied to the cells, which were incubated overnight at 4°C. A diluted biotinylated secondary antibody was then incubated with the sections for 20 min at 37°C. Fresh 3,3-diaminobenzidin (DAB) solution was used to visualize the target proteins, and haematoxylin was used for tissue counterstaining. Two observers independently evaluated target protein expression with an Olympus FV500 optical microscope (Olympus, Tokyo, Japan).

### Statistical analysis

All data are expressed as the mean ± standard deviation; all means were calculated from at least three independent experiments. Statistical significance was determined by a two-tailed Student's t-test or one-way analysis of variance. P<0.05 was considered statistically significant.

## Abbreviations

ADSCs: adipose-derived mesenchymal stem cells
MMP: matrix metalloproteinase
STAT3: signal transducer and activator of transcription 3
TME: tumour microenvironment
MSCs: mesenchymal stem cells
PBS: phosphate-buffered saline
CM: conditioned medium
PI: propidium iodide
